# Economic evaluation of a dietary intervention for adults with major depression (the “SMILES” trial)

**DOI:** 10.1186/s12889-018-5504-8

**Published:** 2018-05-22

**Authors:** Mary Lou Chatterton, Cathrine Mihalopoulos, Adrienne O’Neil, Catherine Itsiopoulos, Rachelle Opie, David Castle, Sarah Dash, Laima Brazionis, Michael Berk, Felice Jacka

**Affiliations:** 1 Deakin University, Deakin Health Economics, Centre for Population Health Research, Waterfront Campus, Room D1.107, Locked Bag 20000, Geelong, VIC 3220 Australia; 2 University of Melbourne, Melbourne School of Population and Global Health, Carlton, Australia; 3 Deakin University, IMPACT Strategic Research Centre, Barwon Health, Geelong, Australia; 4 Centre for Adolescent Health, Murdoch Children’s Research Institute, Melbourne, VIC Australia; 5 Black Dog Institute, Randwick, NSW Australia; 6 Department of Psychiatry, the Florey Institute of Neuroscience and Mental Health, and Orygen Youth Health Research Centre, University of Melbourne, Parkville, VIC Australia; 7 School of Allied Health, La Trobe University, Bundoora, Australia; 8 Department of Psychiatry, University of Melbourne, Melbourne, VIC Australia; 9 Department of Medicine (St Vincent’s campus), The University of Melbourne, Melbourne, Australia

**Keywords:** Depression, Major depressive disorder, Diet, Nutrition, Randomised controlled trial, Economic evaluation

## Abstract

**Background:**

Recently, the efficacy of dietary improvement as a therapeutic intervention for moderate to severe depression was evaluated in a randomised controlled trial. The SMILES trial demonstrated a significant improvement in Montgomery–Åsberg Depression Rating Scale scores favouring the dietary support group compared with a control group over 12 weeks. We used data collected within the trial to evaluate the cost-effectiveness of this novel intervention.

**Methods:**

In this prospective economic evaluation, sixty-seven adults meeting DSM-IV criteria for a major depressive episode and reporting poor dietary quality were randomised to either seven sessions with a dietitian for dietary support or to an intensity matched social support (befriending) control condition. The primary outcome was Quality Adjusted Life Years (QALYs) as measured by the AQoL-8D, completed at baseline and 12 week follow-up (endpoint) assessment. Costs were evaluated from health sector and societal perspectives. The time required for intervention delivery was costed using hourly wage rates applied to the time in counselling sessions. Food and travel costs were also included in the societal perspective. Data on medications, medical services, workplace absenteeism and presenteesim (paid and unpaid) were collected from study participants using a resource-use questionnaire. Standard Australian unit costs for 2013/2014 were applied. Incremental cost-effectiveness ratios (ICERs) were calculated as the difference in average costs between groups divided by the difference in average QALYs. Confidence intervals were calculated using a non-parametric bootstrap procedure.

**Results:**

Compared with the social support condition, average total health sector costs were $856 lower (95% CI -1247 to − 160) and average societal costs were $2591 lower (95% CI -3591 to − 198) for those receiving dietary support. These differences were driven by lower costs arising from fewer allied and other health professional visits and lower costs of unpaid productivity. Significant differences in mean QALYs were not found between groups. However, 68 and 69% of bootstrap iterations showed the dietary support intervention was dominant (additional QALYs at less cost) from the health sector and societal perspectives.

**Conclusions:**

This novel dietary support intervention was found to be likely cost-effective as an adjunctive treatment for depression from both health sector and societal perspectives**.**

**Trial registration:**

Australia and New Zealand Clinical Trials Register (ANZCTR): ACTRN12612000251820. Registered on 29 February 2012.

**Electronic supplementary material:**

The online version of this article (10.1186/s12889-018-5504-8) contains supplementary material, which is available to authorized users.

## Background

Major depressive disorder (MDD) carries a significant burden and cost to health care payers, employers and society [1, 2]. Observational studies have revealed a relationship between diet quality and the risk of depression [3]. However, until recently, this relationship had not been evaluated in the context of a randomised clinical trial (RCT) to allow causality to be elucidated. This led to the creation of the Supporting the Modification of lifestyle In Lowered Emotional States (SMILES) trial, the first RCT to investigate the efficacy of a dietary intervention as an adjunct to treatment of major depressive episodes [4]. The study hypothesized that prescriptive individualised dietary support, focusing on improving diet quality in participants with poor diet quality using a modified Mediterranean diet model [5], would be superior to a social support control condition (befriending) in reducing the severity of depressive symptoms.

The primary results from the SMILES trial demonstrated significant improvement in Montgomery–Åsberg Depression Rating Scale (MADRS) score for the dietary support intervention group compared to the social support control group [6]. A significantly larger proportion of participants in the dietary support group achieved remission versus the control condition, based on MADRS scores.

While the individual cost of providing a dietary support intervention may be perceived as relatively small, indeed there is an opportunity cost associated with any healthcare intervention. This means there is an alternate use for the resources to deliver the dietary support intervention that could be directed toward some other purpose. It is therefore critical to demonstrate the cost-effectiveness of any new interventions to ensure that government and society allocate scarce healthcare dollars where they will have the greatest value-for-money [7].

While dietary counselling has efficacy and cost-effectiveness credentials in the management of obesity, cardiovascular disease and diabetes, it has not previously been evaluated as an adjunctive therapy for the treatment of depression [8]. Therefore, we conducted an economic evaluation within the SMILES RCT to determine whether dietary counselling for people with depression would be cost-effective compared to the social support control condition, where $50,000 per Quality Adjusted Life Year (QALY) is taken as the benchmark for cost-effectiveness in Australia. As recommended by the Second Panel on Cost-effectiveness in Health and Medicine, both health sector and societal perspectives are presented as reference case analyses [9].

## Methods

This economic evaluation utilised data collected as part of the SMILES randomised trial. Details of the SMILES trial protocol and main results have been published elsewhere [4, 6]. In summary, adults meeting the Diagnostic and Statistical Manual of Mental Disorders (4th ed.; DSM-IV-TR) criteria for a major depressive episode (MDE) and poor diet quality using the Dietary Screening Tool were recruited. Exclusion criteria comprised having a concurrent diagnosis of bipolar (type I or II), personality or substance use disorders, two or more failed trials of antidepressants, an unstable medical comorbidity, pregnancy, or a change in medications or psychotherapy in the preceding two weeks. Severe food allergies, intolerances, aversions or socio-cultural reasons for dietary restrictions as well as current participation in an intervention targeting diet or exercise also excluded potential participants. People were recruited in Melbourne and Geelong, Australia between October 2012 and November 2014 using community-based recruitment strategies. Ethical approval was obtained from St. Vincent’s Hospital, Barwon Health and Deakin University Human Research Ethics Committees. All participants provided written informed consent after receiving a full description of the study.

Participants were randomly assigned to the dietary support intervention (the modified Mediterranean diet) [5] or the social support control condition. Personalised dietary advice and nutrition counselling was provided by an Accredited Practicing Dietitian (qualified clinical dietitian) for up to seven individual face-to-face sessions of approximately 60 min each. The social support condition comprised a manualised ‘befriending’ protocol, which has been previously used in psychiatric trials and aims to control for therapeutic effects in RCTs (i.e. time, duration of therapy) [10]. The ‘befriending’ social support condition followed the same visit schedule and length as the dietary support intervention, but consisted of discussion of neutral topics of interest to the participant (i.e. sport, music, news), or in instances where participants found conversation difficult, other activities such as cards or board games. Befriending sessions were guided by a trained research assistant, and the visits did not engage in techniques specifically used in the major therapeutic models of psychotherapy.

### Costs

Australian health sector and societal perspectives were used for the analyses to reflect different decision makers and contexts as per current guidelines for reference cases in economic evaluations [9]. The health sector costs included in this evaluation were those required to deliver the dietary support intervention or the social support control condition, as well as the cost of health care resources used by participants over the trial period. Societal costs comprised the health sector costs in addition to the cost of patient transportation, food and effects on productivity. The cost and outcomes included in the analysis are reported in the Impact Inventory (Additional file 1: Table S1).

The main resource required to deliver both the dietary and the social support was the salary of trained personnel. Detailed records of the number and length of sessions for each participant were collected by dietitians and befrienders. Hourly wage rates with on-costs of 27% were applied to the total time reported to provide the support for each participant. For the dietary support group, the rate was based on a grade two, year one dietitian from the Victorian Hospitals’ Industrial Association salary circular [11]. The hourly wage rate to deliver the social support condition was based on an entry level research assistant salary (Level A, step1) at Deakin University [12]. Since the dietary support intervention aimed to modify the dietary intake of participants, the cost of food was also included in the societal perspective. Food costs were estimated at $112 per week for the dietary support group participants and $138 per week for the social support group participants. This was based on the cost of the first 20 study participants’ dietary intake at baseline compared to the cost of the prescribed healthy alternative, the modified Mediterranean diet [13]. The societal perspective also included transportation costs to attend the dietary or social support sessions. These were estimated by assuming a 30 km round trip in a private vehicle per session, and then applying the cost/km typically used for tax reimbursement in Australia [14].

Participants self-reported the use of prescription medications, over the counter medications and supplements, the number of health professional visits (GPs, psychiatrists, psychologists etc.), and hospitalisations with a resource-use questionnaire administered at baseline and the 12 week follow-up visit. The questionnaire asked for all health care use and was not limited to the use of services specifically for mental health concerns. The health-care costs included in the analyses were those paid by the government as a third-party payer and the out of pocket costs borne by participants, such as co-payments for prescription medications.

Pharmaceutical Benefits Schedule (PBS) data was used to calculate the government and patient out of pocket costs for covered medications [15]. We assumed that all participants would pay the general patient co-payment ($36.10). Online Australian retail pharmacy sites were consulted to determine patient costs for other medications and supplements not covered by the PBS [16–18]. Health professional visits were costed using a weighted average cost paid by the government, derived from the Medicare Benefits Schedule (MBS) item reports [19]. Since a standard co-payment for health professional visits is not in place under the MBS, participants were asked to report the estimated out of pocket costs they paid for these services.

Community mental health visits were costed using data from the 2010 Australian Mental Health Report [20] and the value then inflated to 2013/2014 dollars using the total health price index calculated by the Australian Institute for Health and Welfare [21].

Hospital stays were costed using public sector average cost per separation, based on Australian Refined Diagnosis Related Groups (AR-DRGs) from the National Hospital Cost Data Collection [22]. The specific AR-DRGs were selected based on the reported reason and length of stay.

The societal perspective incorporated patient transportation costs and effects on productivity. Transportation costs for health professional visits were calculated from the distance travelled in kilometres reported in the resource use questionnaire. We assumed a private vehicle was used and therefore multiplied the distance by a cost/km typically used for tax reimbursement [14]. Transport to hospital was estimated based on the cost for a 15 km taxi ride in Melbourne [23].

The resource use questionnaire also asked participants to self-report days off from paid and unpaid work (absenteeism) as well as days worked while suffering health problems as a proxy for presenteeism. To calculate the hours of lost productivity due to presenteeism, we assumed that 1.2 h of productivity was lost per day when a person reported working while suffering health problems [24]. The human capital approach [25] was used to value lost paid productivity by using an average hourly wage rate calculated from the average weekly earnings reported by the Australian Bureau of Statistics plus 25% overhead costs [26]. Time off from unpaid activities (i.e. housework) was valued at 25% of the average wage rate plus overhead costs to represent the value of participants’ lost leisure time [27].

All costs are presented in Australian dollars (AUD) for the 2013/2014 financial reference year. Since the costs and outcomes were collected over a 12 week period, discounting was not applied.

### Outcomes

The primary health outcome for the current study was quality adjusted life years (QALYs). QALYs are useful outcome metrics since they can be applied across different disease/disorder areas, thus allowing comparability across a range of programs when evaluating economic decisions. For example, in Australia $50,000/QALY has been used as a rule of thumb to denote value-for-money [28, 29]. QALYs are derived by “weighing” the length of life spent in a particular health state by the utility or value of that health state. Utility values (or weights) are constrained between 0 and 1 where 0 refers to death and 1 refers to perfect health and values in between denoting less than perfect health states. To assess participants’ health-related quality of life and utility, the Assessment of Quality of Life – Eight Dimension (AQoL-8D) [30] was completed at each assessment. The AQoL-8D is a multi-attribute utility instrument, with a separate utility scoring algorithm associated with the instrument allowing the calculation of utility values for each participant. The utility algorithm used in the current study was derived from the Australian general population [31]. The QALYs for the 12 week follow-up period in the current study were estimated using the area under the curve method [32].

### Statistical analyses

Data management and costing were completed using Excel 2013, while statistical analyses were conducted with Stata 14 (College Station, Texas, USA). SMILES was originally powered to detect differences between groups based on the primary clinical endpoint of MADRS. Therefore, the sample sizes were not designed to test the cost-effectiveness hypotheses. The reference case analyses were undertaken as intention to treat. All enrolled participants who completed a baseline assessment were included; however, 33 % of participants did not complete all of the health care resource use questions at the 12 week follow-up. The missing at random assumption was tested through a series of logistic regression analyses comparing participant characteristics for those with and without missing endpoint data. To account for missing utility values, health care, transportation, and lost productivity costs, the ICE multiple imputation technique in Stata was used [33]. Missing data were imputed 33 times based on the percentage missing from the health care utilisation variables [34]. Generalised linear models (gamma family, log link), using the mim command to combine the estimated coefficients across the imputed datasets, were used to determine the size and significance of differences between groups for total costs (health sector and societal) and QALYs. For each outcome, unadjusted models were developed along with models incorporating the covariates of age, gender, baseline utility and baseline cost.

Incremental cost-effectiveness ratios (ICERs) were calculated as the difference in average cost between the dietary support and social support groups, divided by the difference in average QALYs. Confidence intervals (CIs) for the incremental costs per QALY gained were calculated using a nonparametric bootstrap procedure, with 1000 iterations to reflect the sampling uncertainty. The bootstrapped ICERs and the CIs were graphically represented on cost-effectiveness planes. A cost-effectiveness plane is a plot of the 1000 bootstrapped incremental costs and outcomes across four quadrants. The north-east quadrant represents the intervention costing more as well as conferring greater benefits than the comparator. The south-east quadrant shows the proportion of iterations where the intervention costs less but incurs greater benefits than the comparator (i.e. a “dominant” intervention), the north-west quadrant shows the proportion of iterations where the intervention incurs a cost but fewer benefits than the comparator (i.e. a “dominated” intervention) and, lastly, the south-west quadrant shows the proportion of iterations whereby the intervention costs less and has fewer benefits than the comparator group.

### Sensitivity analyses

Imputation uncertainty was evaluated by comparing the reference case results to the analysis of participants with complete data. Generalised linear models (unadjusted and adjusted) were used to evaluate the size and significance of differences between groups, and non-parametric bootstrapping was also employed concordant with the reference case.

Additional sensitivity analysis was undertaken by varying the cost of the social support control condition to $0 to represent a scenario where the comparator would be a ‘do nothing’ approach. The cost of the dietary support intervention was also varied to assess the effect on the incremental cost difference between groups. Assumptions regarding the food costs were explored by varying the cost of food for the dietary intervention group.

## Results

Demographic characteristics were well balanced between the two study groups at baseline [6]. Additional baseline statistics relevant to the economic evaluation are presented in Table 1. Mean baseline costs (health sector and societal) were greater for the social support group, although only the societal costs were significantly different between groups. However, median values for costs were comparable between the two groups.

**Table 1:** Participant characteristics at baseline

		Total	Dietary Support *n* = 33	Social Support *n* = 34
Age, mean (S.D.) years	mean (S.D.)	40.3 (13.1)	37.4 (10.7)	43.0 (14.6)
Sex, female	% (n)	71.6% (48)	61.8% (21)	81.8% (27)
Post-secondary school education	% (n)	51.5% (34)	51.5% (17)	51.5% (17)
Household income >$80,000/year	% (n)	23.1%(15)	25%(8)	21.2% (7)
Covered by private health insurance	% (n)	50% (32)	50%(16)	50% (16)
Hours of paid work per week	mean (S.D.)	17.7 (17.4)	14.65 (14.9)	20.6 (19.2)
Utility value	mean (S.D.)	0.407 (0.118)	0.390 (0.129)	0.423 (0.104)
Health sector costs^a^	mean (S.D.)	$569 ($1377)	$340 ($391)	$790 ($1881)
	median	$212	$212	$214
Societal costs^a*^	mean (S.D.)	$2228 ($5649)	$1037 ($1438)	$3384 (7683)
	median	$590	$564	$620

^a^costs refer to the month prior to the participant starting the trial

*denotes significant differences at *p* < 0.05

Table 2 provides details of the intervention sessions for both study groups and associated costs. The participants in the dietary support group attended significantly more sessions and had a significantly greater number of contact hours with a dietitian than the participants had with a ‘befriender’. This led to the mean health sector costs for the dietary support group being significantly greater than the social support group. Conversely, the intervention costs from the societal perspective were significantly greater for the social support group due to the additional cost of food.

**Table 2:** Intervention session costs

		Dietary Support	Social Support
Number of sessions*	mean (S.D.)	5.9 (1.6)	4.3 (2.7)
	median	7	5.5
Total contact time (hours)*	mean (S.D.)	4.8 (1.4)	3.4 (2.3)
Health sector cost*	mean (S.D.)	217 (65)	133 (89)
	median	234	148
Societal cost*	mean (S.D.)	1692 (96)	1886 (147)
	median	1711	1934

*denotes significant differences at *p* < 0.05

Note that the societal cost includes food and travel costs

**Fig. 1:**
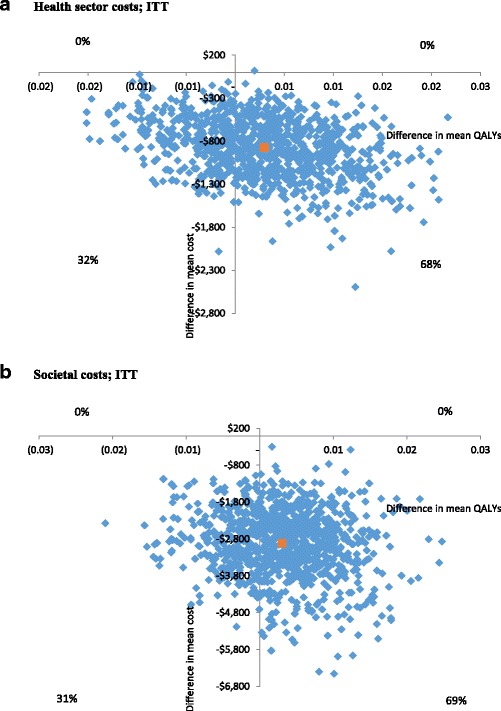
Cost-effectiveness planes. **a** Health sector costs; ITT. **b** Societal costs; ITT

The reference case results using multiple imputation for missing costs, and QALYs analysed with generalised linear models, revealed no significant differences in QALYs between the dietary support and social support groups, even when controlling for baseline age and gender (Table 3). However, significant differences were found for total costs (including the intervention and other costs) from both the health sector and societal perspectives. When the results were transformed from the log scale into dollars, total health sector costs were $856 lower on average, and total societal costs were $2591 lower, for the dietary support group than the social support group. The cost-effectiveness plane in Fig. 1a) shows that 68% of bootstrapped cost-effectiveness ratios using the health sector perspective fell into the south-east quadrant, indicating that the dietary intervention was dominant (lower total costs and more QALYs). Thirty-two percent of iterations fell into the lower left quadrant where the dietary intervention was associated with lower total costs and fewer QALYs. The bootstrapped results from the societal perspective (Fig. 1b) were similar, with 69% of iterations falling into the lower right quadrant, indicating dominance of the dietary support intervention over the social support group, and the remaining 31% falling into the lower cost/fewer QALYs lower left quadrant.

**Table 3:** Effect of the dietary support intervention versus the social support control condition; results are log values

	Model 1: glm (without adjustment)					Model 2: glm (adjusted)^a^				
	Coefficient	Std. Error	95% CI		*P* value	Coefficient	Std. Error	95% CI		*P* value
ITT (imputation used)										
QALYs	0.026	0.063	−0.097	0.150	0.675	0.017	0.066	−0.113	0.147	0.798
Health sector costs	−0.638	0.277	−1.182	−0.093	0.022	−0.605	0.262	−1.119	−0.091	0.021
Societal costs	−0.630	0.205	−1.032	−0.227	0.002	−0.676	0.195	−1.058	−0.293	0.001
Complete Cases										
QALYs	0.036	0.074	−0.108	0.181	0.623	0.039	0.078	−0.113	0.192	0.614
Health sector costs	−0.690	0.289	−1.255	−0.124	0.017	−0.603	0.269	−1.131	−0.075	0.025
Societal costs	−0.675	0.271	−1.206	−0.143	0.013	−0.535	0.249	−1.023	−0.046	0.032

^a^models with QALYs were adjusted for age and gender; cost models were adjusted for age, gender, baseline utility value and baseline cost

Table 4 provides a breakdown of the cost categories comprising total health sector and societal costs. These values were the results of unadjusted generalised linear models that were transformed to represent 2013/2014 Australian dollars, since the log link function produced results on a log scale. The dietary support intervention was associated with significantly greater session delivery and travel costs than the social support group. However, most other cost categories were significantly lower for the dietary support group with the exception of the transportation costs for health care visits. The health care costs were an average of $940 lower, and the cost of lost productivity was $1589 lower, for the dietary support group than the social support group. The social support group used significantly more allied health professionals (occupational therapists, physiotherapists, osteopaths) and other health professionals (dentists, podiatrists, orthodontists) as shown in Additional file 1: Table S2. The results also show that the dietary intervention appeared to reduce productivity costs: that is, participants in the dietary support group missed fewer paid and unpaid work days compared to those in the social support group (Table 4). This difference appears to be largely driven by the significant difference in unpaid productivity costs as demonstrated in Additional file 1: Table S3.

**Table 4:** Total and component cost results transformed from log values; all values are 2013/2014 $AUD

	Social Support	Dietary Support	Mean difference	95% CI	
	mean	mean			
Session delivery	133	217	84	36	145
Health care costs	1684	743	−940	− 1283	− 305
Total health sector costs	1817	960	−856	−1247	−160
Travel for intervention sessions	97	131	35	7	69
Travel for health care	283	103	− 180	− 250	39
Food costs	1656	1344	− 312	−312	−312
Lost productivity costs	2383	794	−1589	− 2102	− 146
Total societal costs	6236	3333	−2591	−3591	−198

### Sensitivity analyses

The analysis of complete cases, defined as participants with complete cost and QALY data at the 12 week follow-up, indicated similar results to the imputed ITT analysis as shown in Table 3. The cost-effectiveness planes (Additional file 1: Figures S1 and S2) show that the dietary intervention was associated with a higher probability of reduced costs and increased QALYS (81 and 80% respectively for health sector and societal perspectives).

The results of the sensitivity analyses are presented in the Additional file 1: Table S4 and S5. When the cost to deliver the social support control condition was adjusted to $0, total health sector costs were no longer significantly different between groups for all ITT results, as well as the complete cases in the adjusted model. The costs from the societal perspective remained significantly different between the study groups, with the exception of the complete case analysis with the adjusted model, where food costs were removed and the social support costs adjusted to $0. When the cost of the dietary intervention increased by a factor of 1.5, the difference in total health sector costs between groups was no longer significant using the adjusted glm (coefficient = − 0.491; *p* = 0.059). As shown in Additional file 1: Table S5, the cost of food for the dietary intervention group would need to increase to $2325 for the dietary intervention to no longer be significant using the results from the base case ITT unadjusted glm model. Using the base case ITT adjusted glm model the dietary intervention was no longer significant when the cost of food increased to $2600. The sensitivity results for the complete cases showed that the results became non-significant at smaller increases in food costs.

## Discussion

This study provides a formal economic evaluation of the first RCT to evaluate dietary improvement as a therapeutic approach for clinical depression. Our findings suggest that a 12 week dietary support intervention had significantly lower costs from both health sector and societal perspectives in the reference case analysis when compared to a control condition. While sensitivity analyses showed this to be fairly robust, results were sensitive to the cost of the comparator to the dietary support intervention. For example, when the cost of the social support intervention were removed, the total health sector costs of dietary support compared to social support were not significantly different. However, the difference remained significant from the societal perspective.

These results suggest that such an intervention has the potential to provide value for money. Nutrition and dietetic services have been shown to be cost-effective for the reduction in LDL cholesterol levels in type 2 diabetes patients [35], and provide a positive return on investment when used to treat patients with hypercholesterolemia [36], as well as other patient groups [37, 38]. Therefore, the current results are in line with the cost-effectiveness of dietary interventions used to treat other health conditions. However, results of analyses of the health care services used by participants demonstrated that the social support group costs were significantly higher for allied and other health professionals, such as dentists, which may appear unrelated to depression. Depression impacts adherence and the therapeutic alliance, and is an adverse prognostic marker across seemingly disparate disorders. However, there is a strong, bidirectional relationship between depression and multiple chronic health conditions, including cardiovascular disease, metabolic syndrome, type 2 diabetes and obesity [39, 40]. Diet quality is an important determinant of all. Fewer visits to non-mental health professionals suggests that a dietary improvement strategy may have multiple benefits that translate to wider health and wellbeing outcomes.

While the utility values for both groups significantly improved over the course of the trial, these values and QALYs were not significantly different between the study groups at 12 week follow-up. The average utility values for this cohort, 0.47 at baseline, were lower than the population norm of 0.83 [41], but similar to the average for individuals with depression from a representative survey of the Australian population [42].

An alternate analytic approach to the current cost-utility analysis would be to use clinical endpoints such as the change in MADRS or symptom remission as outcome measures. However, we did not undertake these analyses since the primary study results demonstrated significant improvement in MADRS scores and a greater percentage of remission in the dietary support intervention over the social support group [6]. Combining these clinical endpoints with the finding that the dietary support intervention was less costly would indicate that the dietary intervention would also be considered the “dominant” strategy providing greater efficacy at a lower cost.

### Strengths and limitations

A strength of the current study was the collection of health care resource utilisation and health preference data prospectively within the trial and the application of conservative unit cost estimates to determine the cost/QALY from a health sector perspective. This evaluation also included a societal perspective as recommended by the Second Panel on Cost-Effectiveness in Health and Medicine [9]. The study design used the social support condition as an active control, which was intended to avoid a nocebo effect that a usual care comparator may induce and to control for the non-specific benefits of face to face interactions [43].

The results of this economic evaluation should, however, be viewed cautiously due to several limitations. The sample size included in the trial was small (Dietary Support group *n* = 33, Social Support group *n* = 34) and limit feasibility and generalisability of the dietary support intervention. Despite the high prevalence of depression in Australia [44], and the presence of poor diet quality [45], few individuals who were interested in participation actually met the entry criteria for the trial. The small sample size, combined with over one-third of missing follow-up cost data and the typically skewed nature of cost data, present additional statistical challenges. While we have attempted to address these using methodologically rigorous methods and conservative assumptions, these results require replication. Moreover, while the outcome assessors were blinded to treatment allocation, trial participants were not. It is unclear whether expectation bias may have influenced the measured outcomes of this analysis in some way. However, psychological support has the greater face validity in the community as an effective intervention.

It is important to note that while there were no significant differences in resource use and costs between the groups at baseline, there were trends toward greater health sector and societal costs in the social support group. However, the adjusted analyses incorporated baseline cost values and showed that the findings were reasonably robust.

Moreover, the resource use and lost productivity data were reliant on participant recall; however, this was over a relatively brief time period (12 weeks). While recall may introduce additional bias into the results, participant-reported data is frequently used in economic evaluations conducted within clinical trials. The brief 12 week follow-up period aids participant recall, however it is unclear whether the cost savings found over the brief time period of this trial would be maintained over the longer term.

The 12 week follow –up may also be perceived as a limitation due to diminishing rates of adherence to diets over longer time periods. However, previous studies evaluating Mediterranean diets for the prevention of cardiovascular disease have shown high rates of adherence to this dietary pattern over two to five years [46–48]. Additionally, given that the cost to deliver the intervention would be incurred up-front (in the first 12 weeks) a longer follow up period may in fact favour the dietary intervention in the cost-effectiveness analysis.

Finally, the resource use questionnaire, while capturing the bulk of service use, did not include questions on all societal costs (e.g. criminal justice costs and community care costs were not explicitly measured). This was largely because brevity of questionnaires was required so that subject recruitment would not be compromised. While it is unlikely that this intervention would affect such costs, we cannot be certain.

## Conclusions

This analysis provides preliminary data to support the cost-effectiveness of a dietary support intervention as an adjunct to medication and psychological care for people with MDE. Further research is needed to validate the results of this novel study.

## Additional file

Additional file 1: **Table S1**. Impact Inventory as recommended by the Second Panel on Cost-Effectiveness in Health and Medicine. **Table S2**. Average costs, contacts and utilisation for health care services for the 3 month follow up period (2013/2014 $AUD). **Table S3**. Average costs, days lost and percent reporting paid and unpaid productivity loss for the 3 month follow up period (2013/2014 $AUD). **Table S4**. Sensitivity analysis results; results are log values. **Table S5**. Sensitivity analysis for food costs; results are log values. **Figure S1**. Cost-effectiveness plane for health sector costs; completers. **Figure S2**. Cost-effectiveness plane for societal costs; completers. **Figure S3**. Cost-effectiveness plane for health sector costs with befriending intervention costs set to $0; ITT. **Figure S4**. Cost-effectiveness plane for societal costs with befriending intervention costs set to $0; ITT. **Figure S5**. Cost-effectiveness plane for health sector costs with befriending intervention costs set to $0; completers. **Figure S6**. Cost-effectiveness plane for societal costs with befriending intervention costs set to $0; completers. (DOCX 249 kb)

## Abbreviations

AQoL-8D: Assessment of Quality of Life – Eight Dimension
AR-DRGs: Australian Refined Diagnosis Related Groups
AUD: Australian dollars
CIs: Confidence intervals
ICER: Incremental cost-effectiveness ratio
MADRS: Montgomery–Åsberg Depression Rating Scale
MBS: Medicare Benefits Schedule
MDD: Major depressive disorder
MDE: Major depressive episode
PBS: Pharmaceutical Benefits Schedule
QALYs: Quality Adjusted Life Years
RCT: Randomised clinical trial
SMILES: Supporting the Modification of lifestyle In Lowered Emotional States
