# Supplementary material for: Economic evaluation of a dietary intervention for adults with major depression (the “SMILES” trial)
Source: BMC Public Health. 2018 May 22;18:599. doi: 10.1186/s12889-018-5504-8 (PMC5963026; doi:10.1186/s12889-018-5504-8)
Supplement: Supplementary file 1 — Table S1. Impact Inventory as recommended by the Second Panel on Cost-Effectiveness in Health and Medicine. Table S2. Average costs, contacts and utilisation for health care services for the 3 month follow up period (2013/2014 $AUD). Table S3. Average costs, days lost and percent reporting paid and unpaid productivity loss for the 3 month follow up period (2013/2014 $AUD). Table S4. Sensitivity analysis results; results are log values. Table S5. Sensitivity analysis for food costs; results are log values. Figure S1. Cost-effectiveness plane for health sector costs; completers. Figure S2. Cost-effectiveness plane for societal costs; completers. Figure S3. Cost-effectiveness plane for health sector costs with befriending intervention costs set to $0; ITT. Figure S4. Cost-effectiveness plane for societal costs with befriending intervention costs set to $0; ITT. Figure S5. Cost-effectiveness plane for health sector costs with befriending intervention costs set to $0; completers. Figure S6. Cost-effectiveness plane for societal costs with befriending intervention costs set to $0; completers. (DOCX 249 kb) [file 12889_2018_5504_MOESM1_ESM.docx]

**Table S1. Impact Inventory as recommended by the Second Panel on Cost-Effectiveness in Health and Medicine**

| Sector | Type of Impact | Included in this reference case analysis from …perspective? | | Notes on sources of evidence |
| --- | --- | --- | --- | --- |
|  |  | Health Care Sector | Societal |  |
| Formal Health Care Sector | | | | |
| Health | Health outcomes (effects) | | | |
|  | Longevity effects |  |  |  |
|  | Health -related quality-of-life effects | ✓ | ✓ | AQoL-8D |
|  | Other health effects (eg, adverse events and secondary transmissions of infections) |  |  |  |
|  | Medical costs | | | |
|  | Paid for by third-party payers | ✓ | ✓ | Self-reported medications, healthcare visits paid by government |
|  | Paid for by patients out-of-pocket | ✓ | ✓ | Self-reported medications, healthcare visits paid by patients |
|  | Future related medical costs (payers and patients) |  |  |  |
|  | Future unrelated medical costs (payers and patients) |  |  |  |
| Informal Health Care Sector | | | | |
| Health | Patient-time costs | NA |  |  |
|  | Unpaid caregiver-time costs | NA |  |  |
|  | Transportation costs | NA | ✓ | Cost per km travelled; self-reported for visits to health care providers; taxi assumed for hospital transport |
| Non-Health Care Sectors (with examples of possible items) | | | | |
| Productivity | Labour market earnings lost | NA | ✓ | Self- reported paid work loss |
|  | Cost of unpaid lost productivity due to illness | NA | ✓ | Self-reported unpaid work loss |
|  | cost of uncompensated household production | NA |  |  |
| Consumption | Future consumption unrelated to health | NA |  |  |
| Social Services | Cost of social services as part of intervention | NA |  |  |
| Legal or criminal justice | Number of crimes related to intervention | NA |  |  |
|  | Cost of crimes related to intervention | NA |  |  |
| Education | Impact of intervention on educational achievement of population | NA |  |  |
| Housing | Cost of intervention on home improvements (eg, removing lead paint) | NA |  |  |
| Environment | production of toxic waste pollution by intervention | NA |  |  |
| Other (specify) | Food costs | NA | ✓ | Estimated cost from detail of food diary of first 20 participants at baseline |

Template based on Figure 1 from Sanders et al, JAMA 2016.

**Table S2. Average costs, contacts and utilisation for health care services for the 3 month follow up period (2013/2014 $AUD)**

|  | **Social support** | | |  |  |  | **Dietary support** | | |  |  |  |
| --- | --- | --- | --- | --- | --- | --- | --- | --- | --- | --- | --- | --- |
|  | **mean** | **SD** | **Percentage of total cost** | **Mean contacts** | **SD** | **Using service** | **mean** | **SD** | **Percentage of total cost** | **Mean contacts** | **SD** | **Using service** |
| Medications, supplements* | 82 | 99 | 5.1 | 1.8 | 1.4 | 95.0% | 169 | 164 | 22.2 | 2.6 | 1.6 | 85.7% |
| Hospitalisations | 436 | 1622 | 27.2 | 0.22 | 0.67 | 13.4% | 89 | 513 | 11.7 | 0.04 | 0.19 | 37.0% |
| GP | 98 | 104 | 6.1 | 1.9 | 1.6 | 82.6% | 95 | 98 | 12.4 | 1.7 | 1.9 | 70.4% |
| Nurse | 1 | 3 | 0.0 | 0.0 | 0.2 | 4.6% | 1 | 3 | 0.1 | 0.0 | 0.2 | 3.7% |
| Psychiatrist | 147 | 296 | 9.2 | 0.9 | 1.8 | 30.4% | 88 | 228 | 11.5 | 0.4 | 1.0 | 14.8% |
| Psychologist | 270 | 514 | 16.9 | 1.8 | 3.7 | 26.1% | 151 | 276 | 19.8 | 1.3 | 2.6 | 40.7% |
| Social worker | 0 | 0 | 0.0 | 0.0 | 0.0 | 0.0% | 18 | 68 | 2.4 | 0.2 | 0.9 | 8.0% |
| Community health centre | 49 | 159 | 3.1 | 0.2 | 0.8 | 9.5% | 80 | 406 | 10.4 | 0.4 | 2.0 | 3.9% |
| Allied health professional* | 376 | 1059 | 23.4 | 2.1 | 3.8 | 31.8% | 48 | 90 | 6.3 | 0.4 | 0.8 | 26.9% |
| Other health professionals* | 144 | 305 | 9.0 | 1.4 | 4.3 | 31.8% | 24 | 88 | 3.1 | 0.2 | 0.5 | 15.4% |
|  |  |  |  |  |  |  |  |  |  |  |  |  |

*indicates significant differences between groups in mean costs

The sum of the mean costs of health care services does not total the average total costs. This is because the main results use multiple imputation for total healthcare costs while non-imputed data was used to construct this table.

**Table S3. Average costs, days lost and percent reporting paid and unpaid productivity loss for the 3 month follow up period (2013/2014 $AUD)**

|  | **Social support** | | | |  |  |  | **Dietary support** | | |  |  |  |
| --- | --- | --- | --- | --- | --- | --- | --- | --- | --- | --- | --- | --- | --- |
|  | **Mean** | **SD** | **Percentage of total cost** | | **Mean days off** | **SD** | **Reporting days off** | **Mean** | **SD** | **Percentage of total cost** | **Mean days off** | **SD** | **Reporting days off** |
| Paid productivity | 1783 | 3826 | 65% | 1.3 | | 3.4 | 21% | 661 | 1583 | 71% | 0.5 | 1.5 | 14% |
| Unpaid productivity* | 939 | 1864 | 35% | 3.0 | | 6.6 | 41% | 274 | 557 | 29% | 0.8 | 1.9 | 20% |

*indicates significant differences between groups in mean costs

**Table S4. Sensitivity analysis results; results are log values**

|  | Model 1: glm (without adjustment) | | | | | |  | Model 2: glm (adjusted)* | | |  |  |
| --- | --- | --- | --- | --- | --- | --- | --- | --- | --- | --- | --- | --- |
|  | Coefficient | | Std. Error | | 95% CI | | P value | Coefficient | Std. Error | 95% CI | | P value |
| **ITT (imputation used)** | | |  |  | |  |  |  |  |  |  |  |
| **Social support costs = $0** | | |  |  | |  |  |  |  |  |  |  |
| Health sector costs | -0.561 | | 0.293 | -1.138 | | 0.016 | 0.057 | -0.512 | 0.280 | -1.062 | 0.038 | 0.068 |
| Societal costs | -0.608 | | 0.208 | -1.017 | | -0.199 | 0.004 | -0.653 | 0.197 | -1.041 | -0.265 | 0.001 |
| **Food costs removed** | |  |  |  | |  |  |  |  |  |  |  |
| Societal costs | -0.837 | | 0.303 | -1.432 | | -0.242 | 0.006 | -0.879 | 0.294 | -1.456 | -0.302 | 0.003 |
| **Social support = $0 & food costs removed** | | | |  | |  |  |  |  |  |  |  |
| Societal costs | -0.808 | | 0.308 | -1.413 | | -0.202 | 0.009 | -0.844 | 0.299 | -1.431 | -0.256 | 0.005 |
| **Completers** |  | |  |  | |  |  |  |  |  |  |  |
| **Social support costs = $0** | | |  |  | |  |  |  |  |  |  |  |
| Health sector costs | -0.604 | | 0.306 | -1.204 | | -0.004 | 0.048 | -0.490 | 0.289 | -1.056 | 0.076 | 0.090 |
| Societal costs | -0.651 | | 0.275 | -1.190 | | -0.113 | 0.018 | -0.504 | 0.252 | -0.997 | -0.011 | 0.045 |
| **Food costs removed** | | |  |  | |  |  |  |  |  |  |  |
| Societal costs | -0.877 | | 0.393 | -1.648 | | -0.106 | 0.026 | -0.612 | 0.373 | -1.344 | 0.120 | 0.101 |
| **Social support = $0 & food costs removed** | | | |  | |  |  |  |  |  |  |  |
| Societal costs | -0.846 | | 0.399 | -1.629 | | -0.064 | 0.034 | -0.553 | 0.379 | -1.296 | 0.190 | 0.144 |

*models were adjusted for age, gender, baseline utility value and baseline cost

**Table S5. Sensitivity analysis for food costs; results are log values**

|  | Model 1: glm (without adjustment) | | | |  | Model 2:glm (adjusted)* | | | |  | |  | |  |
| --- | --- | --- | --- | --- | --- | --- | --- | --- | --- | --- | --- | --- | --- | --- |
|  | Coefficient | Std. Error | 95% CI | | P value | Coefficient | Std. Error | | 95% CI | | | | P value | |
| **ITT (imputation used)** | |  |  |  |  |  |  | |  | |  | |  | |
| **Food costs equal between groups ($1656)** | | | | | |  | |  | |  | |  | |  |
| Societal costs | -0.540 | 0.199 | -0.931 | -0.149 | 0.007 | -0.583 | 0.188 | | -0.953 | | -0.213 | | 0.002 | |
| **Food costs =$2325 for dietary support** | | |  |  |  |  |  | |  | |  | |  | |
| Societal costs | -0.372 | 0.190 | -0.746 | 0.002 | 0.051 | -0.410 | 0.178 | | -0.760 | | -0.059 | | 0.022 | |
| **Food costs =$2600 for dietary support** | | |  |  |  |  |  | |  | |  | |  | |
| Societal costs | -0.310 | 0.188 | -0.679 | 0.058 | 0.099 | -0.346 | 0.175 | | -0.691 | | -0.002 | | 0.049 | |
| **Completers** |  |  |  |  |  |  |  | |  | |  | |  | |
| **Food costs equal between groups ($1656)** | | | | | |  | |  | |  | |  | |  |
| Societal costs | -0.591 | 0.261 | -1.103 | -0.079 | 0.024 | -0.446 | 0.239 | | -0.915 | | 0.023 | | 0.062 | |
| **Food costs =$2050 for dietary support** | | |  |  |  |  |  | |  | |  | |  | |
| Societal costs | -0.494 | 0.252 | -0.988 | -0.001 | 0.050 | -0.345 | 0.229 | | -0.794 | | 0.105 | | 0.133 | |

*models were adjusted for age, gender, baseline utility value and baseline cost

**Figure S1. Cost-effectiveness plane for health sector costs; completers**

12%

0%

81%

7%

**Figure S2. Cost-effectiveness plane for societal costs; completers**

13%

%%

0%

%%

81%

%%

9.5%

%%

**Figure S3. Cost-effectiveness plane for health sector costs with befriending intervention costs set to $0; ITT**

35%

65%

0%

**Figure 4S. Cost-effectiveness plane for societal costs with befriending intervention costs set to $0; ITT**

0%

0%

31%

69%

**Figure S5. Cost-effectiveness plane for health sector costs with befriending intervention costs set to $0; completers**

8%

18%

0%

**Figure S6. Cost-effectiveness plane for societal costs with befriending intervention costs set to $0; completers**

0%

12%

8%

80%
